# Meta-DiSc: a software for meta-analysis of test accuracy data

**DOI:** 10.1186/1471-2288-6-31

**Published:** 2006-07-12

**Authors:** Javier Zamora, Victor Abraira, Alfonso Muriel, Khalid Khan, Arri Coomarasamy

**Affiliations:** 1 Clinical Biostatistics Unit, Ramón y Cajal Hospital, Madrid, Ctra. Colmenar km 9.100 Madrid 28034, Spain; 2 University of Birmingham and Birmingham Women's Hospital, Edgbaston, Birmingham, UK

## Abstract

**Background:**

Systematic reviews and meta-analyses of test accuracy studies are increasingly being recognised as central in guiding clinical practice. However, there is currently no dedicated and comprehensive software for meta-analysis of diagnostic data. In this article, we present Meta-DiSc, a Windows-based, user-friendly, freely available (for academic use) software that we have developed, piloted, and validated to perform diagnostic meta-analysis.

**Results:**

Meta-DiSc a) allows exploration of heterogeneity, with a variety of statistics including chi-square, I-squared and Spearman correlation tests, b) implements meta-regression techniques to explore the relationships between study characteristics and accuracy estimates, c) performs statistical pooling of sensitivities, specificities, likelihood ratios and diagnostic odds ratios using fixed and random effects models, both overall and in subgroups and d) produces high quality figures, including forest plots and summary receiver operating characteristic curves that can be exported for use in manuscripts for publication. All computational algorithms have been validated through comparison with different statistical tools and published meta-analyses. Meta-DiSc has a Graphical User Interface with roll-down menus, dialog boxes, and online help facilities.

**Conclusion:**

Meta-DiSc is a comprehensive and dedicated test accuracy meta-analysis software. It has already been used and cited in several meta-analyses published in high-ranking journals. The software is publicly available at .

## Background

Accurate diagnosis forms the basis of good clinical care, as without it one can neither prognosticate correctly nor choose the right treatment. Indeed, a wrong diagnosis can harm patients by exposing them to inappropriate or sub-optimal therapy [1]. Thus studies of diagnostic accuracy, and particularly their systematic reviews and meta-analyses, are being recognised as instrumental in underpinning evidence-based clinical practice. Initiatives such as STARD [2] and developments within the Cochrane Collaboration [3] to accept protocols and reviews of test accuracy studies highlight the emphasis being given to evidence-based diagnosis.

Currently, there is only one test accuracy meta-analysis package, Meta-Test [4], which addresses some of the unique statistical issues related to test accuracy, such as pooling of sensitivities and specificities and summary receiver operating characteristics (sROC) analysis. However, it is a DOS-based application with an interface that many find difficult to use, and integrate into Windows-based applications. Moreover, it lacks crucial analytical tools such as pooling of likelihood ratios (LRs), tests for heterogeneity and meta-regression facilities.

We, therefore, developed, piloted and validated a comprehensive, Windows-based test accuracy meta-analysis software, Meta-DiSc, which is presented in this article, with a worked example.

## Implementation

Meta-DiSc software was created in Microsoft Visual Basic 6, and some mathematical routines have been linked from the NAG C mathematical library [5]. The software is distributed as a single file, downloadable freely from URL: . Its installation is simple, guided by onscreen instructions. The programme has a user-friendly interface with roll-down menus, dialog boxes and online HTML compiled help files. These help files include a user manual and a description of the implemented statistical methods.

Meta-DiSc allows data entry into its datasheet in three different ways: a) directly by typing data into the datasheet using the keyboard, b) copying from another spreadsheet (e.g. Microsoft Excel) and pasting into Meta-DiSc datasheet, or c) importing text files from other sources (for example, in the comma delimited format). Several variables can be defined in the datasheet, including study identifiers, accuracy data from each study (true positives, false positives, true negatives and false negatives) and study level co-variates, such as those defining population spectrum or methodological quality of the studies.

**Figure 1:**
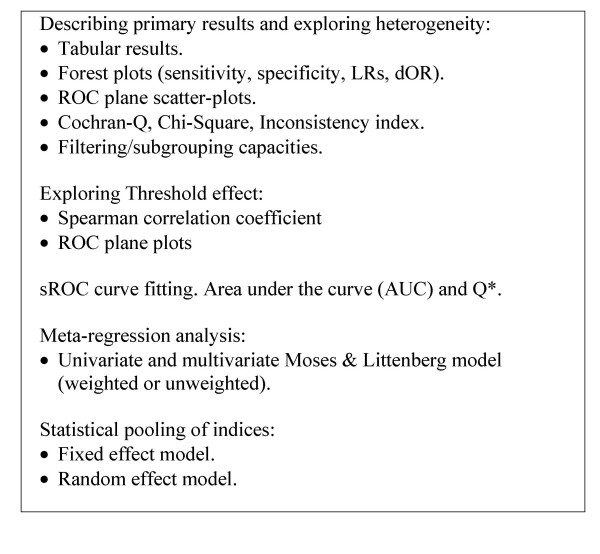
**Available tools in Meta-DiSc**. Tools implemented in the software Meta-DiSc to perform different steps of meta-analysis of diagnostic tests accuracy.

Once the data have been entered into the datasheet of Meta-DiSc, various statistical analyses can be implemented (Figure 1). The implementation of these statistical procedures needs to be carefully thought through and judicious, as it may be inappropriate (or indeed misleading) to use all the procedures (particularly statistical pooling) in all reviews. Meta-DiSc provides analysts with adequate tools to assess the appropriateness of pooling. Readers interested in details of these methods are referred to statistical methods section of the help files (also available as a PDF standalone document [6] and to existing texts and guidelines on diagnostic meta-analysis [7-10].

### Describing the results of individual studies

When describing accuracy results from several studies, it is important to get an indication of the magnitude and precision of the accuracy estimates derived from each study, as well as to assess the presence or absence of inconsistencies in accuracy estimates across studies (heterogeneity). As accuracy estimates are paired and often inter-related (sensitivity and specificity, or LR positive and LR negative), it is necessary to report these simultaneously [11]. One accuracy measure that combines these paired measures is diagnostic odd ratio (dOR) [12], which has limited clinical use, although useful in procedures like meta-regression (see below).

Meta-DiSc computes accuracy estimates and confidence intervals from individual studies and shows results either as numerical tabulations or graphical plots in two formats: a) forest plots, for sensitivities, specificities, LRs or dOR, with respective confidence intervals; and b) plots of individual study results in ROC space, with or without an sROC curve.

### Exploring heterogeneity (threshold effect)

Exploring heterogeneity is a critical issue to a) understand the possible factors that influence accuracy estimates, and b) to evaluate the appropriateness of statistical pooling of accuracy estimates from various studies. One of the primary causes of heterogeneity in test accuracy studies is threshold effect, which arises when differences in sensitivities and specificities or LRs occur due to different cut-offs or thresholds used in different studies to define a positive (or negative) test result. When threshold effect exists, there is a *negative *correlation between sensitivities and specificities (or a *positive *correlation between sensitivities and 1-specificities), which results in a typical pattern of "shoulder arm" plot in a sROC space [8]. It is worth noting that correlation between sensitivity and specificity could arise due to a number of reasons other than threshold (e.g. partial verification bias, different spectrum of patients or different settings).

Meta-DiSc allows assessment for threshold effect in three different ways: a) visual inspection of relationship between pairs of accuracy estimates in forest plots. If threshold effect is present, the forest plots will show increasing sensitivities with decreasing specificities, or vice versa. The same inverse relationship will be apparent with LR positive and LR negative; b) representation of accuracy estimates from each study in a sROC space – a typical "shoulder arm" pattern would suggest presence of threshold effect; and c) computation of Spearman correlation coefficient between the logit of sensitivity and logit of 1-specificity. A strong *positive *correlation would suggest threshold effect.

### Exploring for heterogeneity (other than threshold effect)

Apart from variations due to threshold effect, there are several other factors that can result in variations in accuracy estimates amongst different test accuracy studies in a review. These reasons include chance as well as variations in study population (e.g. severity of disease and co-morbidities), index test (differences in technology, assays, operator etc.), reference standard, and the way a study was designed and conducted [13]. Since such heterogeneity is almost always present in accuracy systematic reviews, testing for the presence and the extent of heterogeneity of results between primary studies, prior to undertaking any meta-analysis, is a critical part of any diagnostic review, as is exploration of the possible causes of heterogeneity [14].

Meta-DiSc allows users to test for heterogeneity amongst various studies in two different ways: a) Visual inspection of forest plots of accuracy estimates. If the studies are reasonably homogeneous, the accuracy estimates from individual studies will lie along a line corresponding to the pooled accuracy estimate. Large deviations from this line will indicate possible heterogeneity; b) statistical tests, including Chi-square and Cochran-Q, which are automatically implemented during analysis to evaluate if the differences across the studies are greater than expected by chance alone. A low p-value will suggest presence of heterogeneity beyond what could be expected by chance alone. In addition to these heterogeneity statistics, Meta-DiSc computes the inconsistency index (I-squared) which has been proposed as a measure to quantify the amount of heterogeneity [15].

### Meta-regression

If substantial heterogeneity is found to be present from the analyses detailed above, then reasons for such heterogeneity can be explored by relating study level co-variates (e.g., population, test, reference standard or methodological features) to an accuracy measure, using meta-regression techniques. The accuracy measure that is normally used is dOR, as it is a unitary measure of diagnostic performance that encompasses both sensitivity and specificity or both LR positive and LR negative. Using dOR as a global measure of accuracy is a suitable method to compare the overall diagnostic accuracy of different tests [13]. However, its use is limited because it cannot be used directly in clinical practice and, furthermore, possible opposing effects of a study characteristic on sensitivity or specificity may be masked by using dOR.

Meta-DiSc implements meta-regression using a generalization of Littenberg and Moses Linear model [8,13] weighted by inverse of the variance or study size or unweighted. Random effects between studies can be estimated by different methods and added to the weighting scheme [16]. Estimations of coefficients of the model are performed by least squares method as implemented in NAG mathematical routines. The outcome variable is ln(dOR) which is related via a linear model to any number of study level covariates, and optionally including the variable representing threshold effect [13]. The outputs from meta-regression modelling in Meta-DiSc are the co-efficients of the model, as well as ratio of dOR (rdOR) with respective confidence intervals. If a particular study level co-variate is significantly associated with diagnostic accuracy, then its co-efficient will have a low p-value, and the rdOR will give a measure of magnitude of the association.

More advanced meta-regression techniques such as Hierarchical sROC model [17] and bivariate analysis of sensitivity and specificity [18] has been developed. These methods overcome some of the statistical shortcomings inherent to Littenberg and Moses model [8,19].

### Statistical pooling

Statistical pooling is not always appropriate or necessary in every systematic review of test accuracy studies. However, when used appropriately, pooling can provide useful summary information. The necessary precondition for simple pooling (weighted averaging) of each of sensitivities, specificities, LR positives and LR negatives, is that the studies and results are reasonably homogeneous (i.e. no substantial heterogeneity, including threshold effect, is present). If heterogeneity due to threshold effect were present, the accuracy data can be pooled by fitting a sROC curve and summarising that curve by means of the Area Under the Curve (AUC) or using other statistics such as the Q* index [19] (i.e. the point of the curve in which sensitivity equals specificity). If there is heterogeneity due to sources other than threshold effect, then pooling should only be attempted within homogeneous subsets, which would normally have been defined a priori.

Meta-DiSc has comprehensive functionality for statistical pooling: a) It allows pooling of sensitivities, specificities, LR positive and LR negative each separately, using either fixed or random effect [10,20] models. The output from these analyses are presented numerically in tables, and graphically as forest plots. Pooled estimates are provided with their respective confidence intervals; b) It implements several ways to fit a sROC curve when threshold effect is present. Default option is to compute a symmetrical sROC curve after fitting the linear model proposed by Littenberg and Moses. However, users can choose different options to fit this curve, for example, combining individual *dORs *by the Mantel-Haenszel or the DerSimonian Laird methods [10,20] to estimate an overall *dOR*, and then fitting an sROC curve. When the *dOR *changes with diagnostic threshold, the sROC curve is asymmetrical. Meta-DiSc allows the user to check for asymmetry of the sROC curve, and fit an asymmetrical sROC curve if appropriate. Finally, Meta-DiSc allows estimation of AUC and the Q* index, along with their standard errors, as a summary measure of global accuracy which also aids inter-test comparisons; c) Meta-DiSc allows pooling of various summary measures within subgroups defined by study level co-variates with the help of a filter utility.

Wherever possible, the results of the above statistical procedures were validated using different general purpose statistical software such as STATA (ver 8.2) and SAS (8.2) using actually published and simulated data sets (Table 1).

**Table 1:** Validation of statistical procedures. Validation of different statistical procedures using a simulated data-set. Results of Meta-DiSc (version 1.4) are compared with those obtained with metan (version 1.86) and metareg (version 1.06) STATA commands. Prior to the analyses, all four cells of all studies were added with 1/2 to avoid division by zero when computing some indices or standard errors. Meta-DiSc and STATA data-set are provided as additional files [see Additional file 1] and [see Additional file 2].

	Results	
	
Procedure	Meta-DiSc (version 1.4)	STATA (ver 8.2)
Random Effect Model		
Pooled +ve LR	2.447	2.447
(95%(CI)	(2.085 – 2.871)	(2.085 – 2.871)
Tau-square	0.0932	0.0932
Cochrane-Q	139.71	139.71
		
Pooled -ve LR	0.157	0.157
(95%(CI)	(0.095 – 0.257)	(0.095 – 0.257)
Tau-square	0.4631	0.46357
Cochrane-Q	33.00	33.07
		
Fixed Effect Model		
Pooled +ve LR	2.330	2.330
(95%(CI)	(2.208 – 2.459)	(2.208 – 2.459)
Cochrane-Q	139.71	139.71
		
Pooled -ve LR	0.105	0.104
(95%(CI)	(0.073 – 0.149)	(0.073 – 0.148)
Cochrane-Q	33.00	33.07
		
Meta-Regression^1^		
Tau-Square	0.1141	0.1141
Constant coefficient (SE)	2.520 (0.8370)	2.5197 (0.83699)
S coefficient (SE)	0.330 (0.1912)	0.3304 (0.19123)
Covariable coefficient (SE)	-0.036 (0.0904)	-0.0355 (0.09041)

(1) Meta-regression was weighted by the inverse of the variance of dOR and between study variance was estimated by REML.

## Results

We illustrate the various procedures that Meta-DiSc implements in a case-study of ultrasound test in the diagnosis of uterine pathology [21,22]. Ultrasound measurement of the lining of the uterus (endometrium) can predict pathology such as endometrial hyperplasia (a precancerous condition) or cancer. The greater the thickness of endometrium, the more likely that the target condition is present. Various thresholds (such as 3, 4 or 5 mm etc) have been used to define a positive ultrasound result.

**Figure 2:**
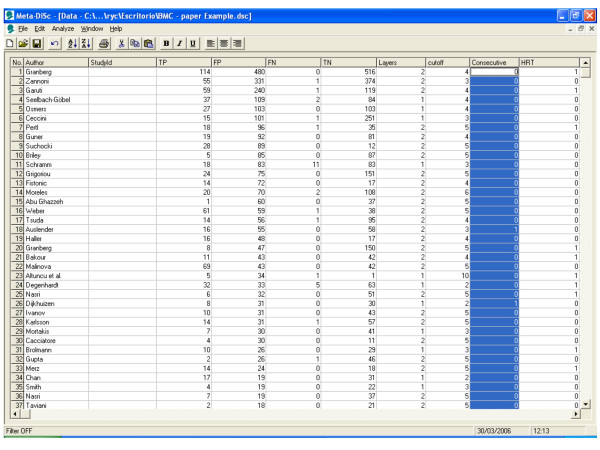
**Meta-Disc datasheet**. Meta-DiSc data set with details of test accuracy studies of ultrasound in the prediction of endometrial cancer.

A systematic review of test accuracy studies identified 57 studies. Figure 2 shows a datasheet in Meta-DiSc which has been loaded with information from these 57 studies. The information includes study identifiers, accuracy data, thresholds, and some study level co-variates (such as hormone replacement therapy use).

**Figure 3:**
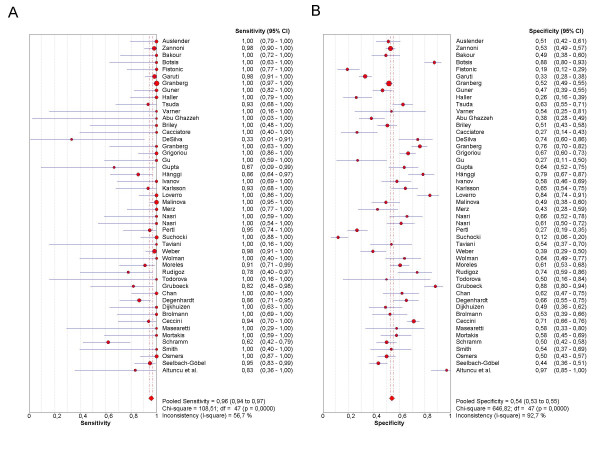
**Forest plot**. Forrest plot of sensitivities (3a) and specificities (3b) from test accuracy studies of ultrasound in the prediction of endometrial cancer.

**Figure 4:**
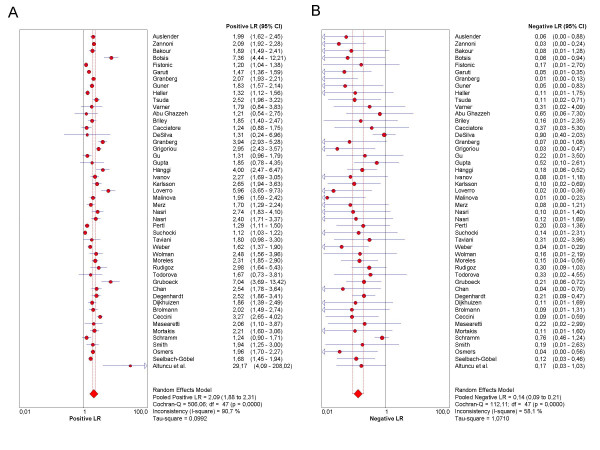
**Forest plot**. Forrest plot of likelihood ratios for positive (4a) and negative (4b) test results from studies of ultrasound in the prediction of endometrial cancer.

**Figure 5:**
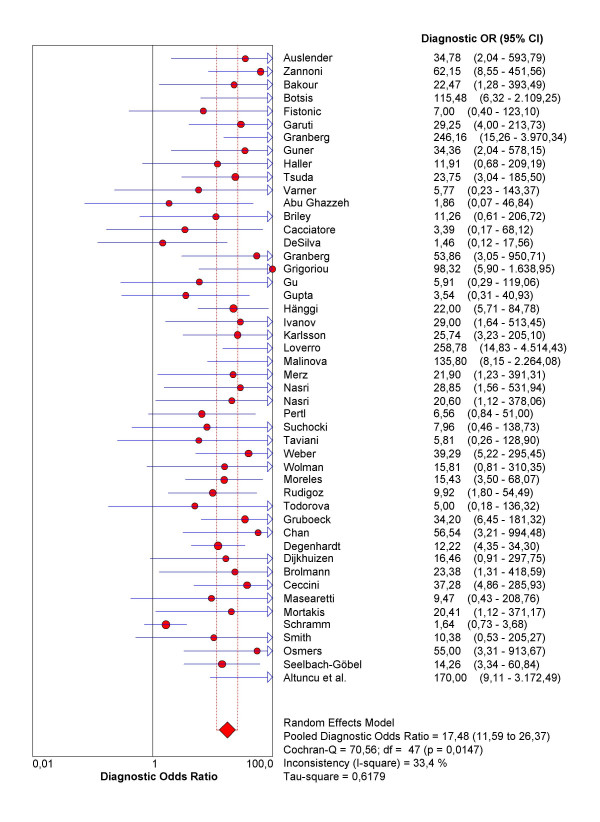
**Forrest plot**. Forest plot of diagnostic odds ratios (dOR) from test accuracy studies of ultrasound in the prediction of endometrial cancer.

As the first step in the analysis, we have used Meta-DiSc to present accuracy measures from each individual study in forest plots for sensitivities (figure 3a), specificities (figure 3b), LRs (figures 4a and 4b) and dOR (figure 5). All these indices can also be represented in tabular form as shown in table 2. Although the forest plots and the tables contain a pooled summary at the bottom, at this early stage in the analysis, it is recommended that the plots are used to obtain a general overview of the accuracy estimates from each study, and the interpretation of the pooled summary is left to later stages of analysis.

**Table 2:** Tabulation of Likelihood ratio for positive test result (LR+) with respective 95% confidence intervals from all test accuracy studies included in systematic review of ultrasound for prediction of endometrial cancer.

Study	LR+	[95% Conf. Iterval.]		% Weight
Auslender	1,994	1,623	-2,449	2,54
Zannoni	2,092	1,919	-2,280	2,77
Bakour	1,895	1,490	-2,408	2,45
Botsis	7,360	4,437	-12,208	1,69
Fistonic	1,200	1,045	-1,378	2,69
Garuti	1,471	1,358	-1,593	2,78
Granberg	2,066	1,935	-2,206	2,79
Guner	1,834	1,569	-2,144	2,65
Haller	1,321	1,118	-1,561	2,63
Tsuda	2,517	1,964	-3,225	2,43
Varner	1,795	0,842	-3,826	1,13
Abu Ghazzeh	1,215	0,538	-2,745	1,03
Briley	1,855	1,396	-2,465	2,33
Cacciatore	1,239	0,877	-1,752	2,15
DeSilva	1,306	0,245	-6,957	0,34
Granberg	3,937	2,933	-5,284	2,30
Grigoriou	2,946	2,430	-3,572	2,57
Gu	1,307	0,956	-1,787	2,25
Gupta	1,846	0,783	-4,350	0,96
Hänggi	4,000	2,472	-6,473	1,76
Ivanov	2,273	1,691	-3,054	2,30
Karlsson	2,649	1,936	-3,627	2,24
Loverro	5,957	3,648	-9,729	1,73
Malinova	1,963	1,591	-2,421	2,53
Merz	1,697	1,287	-2,236	2,35
Nasri	2,740	1,833	-4,096	1,98
Nasri	2,400	1,711	-3,367	2,17
Pertl	1,293	1,115	-1,499	2,67
Suchocki	1,120	1,027	-1,222	2,77
Taviani	1,802	0,983	-3,304	1,44
Weber	1,618	1,374	-1,904	2,64
Wolman	2,481	1,556	-3,956	1,80
Moreles	2,312	1,845	-2,896	2,49
Rudigoz	2,981	1,638	-5,426	1,46
Todorova	1,667	0,729	-3,808	1,01
Gruboeck	7,036	3,689	-13,422	1,35
Chan	2,543	1,779	-3,635	2,12
Degenhardt	2,516	1,856	-3,411	2,27
Dijkhuizen	1,859	1,389	-2,489	2,31
Brolmann	2,017	1,487	-2,736	2,27
Ceccini	3,267	2,655	-4,021	2,54
Masearetti	2,059	1,096	-3,866	1,38
Mortakis	2,213	1,602	-3,058	2,22
Schramm	1,241	0,899	-1,714	2,22
Smith	1,938	1,252	-3,001	1,88
Osmers	1,964	1,699	-2,271	2,68
Seelbach-Göbel	1,680	1,455	-1,940	2,68
Altuncu et al.	29,167	4,089	-208,02	0,25

**(REM) pooled LR+**	**2,087**	**1,881**	**-2,315**	

Heterogeneity chi-squared = 506,06 (d.f.= 47) p = 0,000

Inconsistency (I-square) = 90,7%

No. studies = 48.

Filter OFF

Add 1/2 only zero cell studies

**Figure 6:**
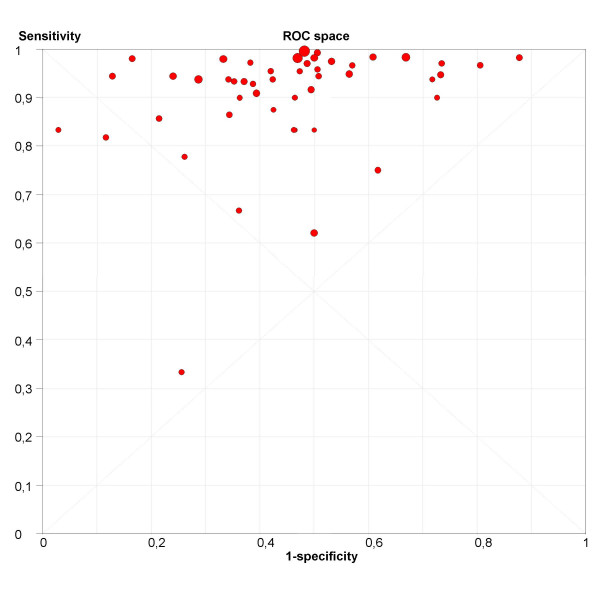
**ROC Space**. Representation of sensitivity against (1-specificity) in Receiver Operating Characteristics space for each study of ultrasound in the prediction of endometrial cancer.

The next step is the representation of sensitivity against 1-specificity from each study in a ROC space (figure 6), which can be used for exploration for threshold effect. The pattern of the points in this plot suggest a "shoulder-arm" shape, indicating the possibility of threshold effect. We, therefore, performed a Spearman rank correlation as a further test for threshold effect, and found that there was further indication of threshold effect (Table 3, Spearman correlation coefficient = 0.394; p = 0.006). Having found some clues about the presence of threshold effect, we now focus on a subgroup of 21 studies that used a singular threshold of >5 mm to define test positivity. Although an *explicit *threshold of 5 mm was used in these studies, there can still be an *implicit *threshold effect due to, for example, variation in the interpretation of the test results. Therefore, within this subgroup with an explicit threshold of 5 mm, it is still recommended that the above explorations for threshold effect are undertaken. We performed such analyses for this subgroup in Meta-DiSc, and found no evidence of further threshold effect (data not shown). There are a number of other more advanced methods not implemented in Meta-DiSc that allow to incorporate explicitly information about tests thresholds defined between or within studies [17].

**Table 3:** Results of Spearman rank correlation of sensitivity against (1 – specificity) to assess the threshold effect in all test accuracy studies included in systematic review of ultrasound for prediction of endometrial cancer.

Var.	Coeff.	Std. Error	T	p-value
A	2.412	0.292	8.266	0.0000
b(1)	0.187	0.101	1.857	0.0697

Spearman correlation coefficient: 0,394 p-value = 0,006 (Logit(TPR) vs Logit(FPR)

Moses' model (D = a + bS)

Unweighted regression

Tau-squared estimate = 0,3540

(Convergence is achieved after 2 iterations)

Restricted Maximum Likelihood estimation (REML)

No. studies = 48

Filter OFF

As the next step, heterogeneity arising from factors other than threshold effect is explored. We performed a visual exploration of the forest plots of accuracy measures for these 21 studies as well as statistical tests for heterogeneity (Meta-DiSc output not shown). In addition, possible sources of heterogeneity across the studies were explored using meta-regression analysis with the following co-variates as predictor variables: use or non-use of hormone replacement therapy (HRT); technique of ultrasound measurement (single or double layer); and population enrolment (consecutive or other). Results are shown in Table 4, which suggest that the number of layers is strongly associated with accuracy. The double layer technique is associated with two times higher accuracy compared to single layer measurement (rdOR = 2.04; 95% CI: 1.01–4.13; p = 0.048)

**Table 4:** Results of meta-regression analysis for predicting the presence or absence of endometrial carcinoma with variables: use or non-use of hormone replacement therapy (HRT); technique of ultrasound measurement (single or double layer); and population enrolment (consecutive or other).

**Meta-Regression(Inverse Variance weights) (1)**				
Var.	Coeff.	p-value	RDOR	[95%CI]
Cte.	0,857	0,1571	----	----
S	0,263	0,0208	----	----
Layers	0,709	0,0610	2,03	(0,97;4,27)
Consecutive	0,206	0,7398	1,23	(0,35;4,26)
HRT	0,324	0,4152	1,38	(0,63;3,06)
				
**Meta-Regression(Inverse Variance weights) (2)**				
Var.	Coeff.	p-value	RDOR	[95%CI]

Cte.	0,849	0,1565	----	----
S	0,253	0,0194	----	----
Layers	0,739	0,0424	2,09	(1,03;4,27)
HRT	0,320	0,4152	1,38	(0,63;3,02)
				
**Meta-Regression(Inverse Variance weights) (3)**				
Var.	Coeff.	p-value	RDOR	[95%CI]

Cte.	0,959	0,0999	----	----
S	0,258	0,0166	----	----
Layers	0,712	0,0482	2,04	(1,01;4,13)

**Figure 7:**
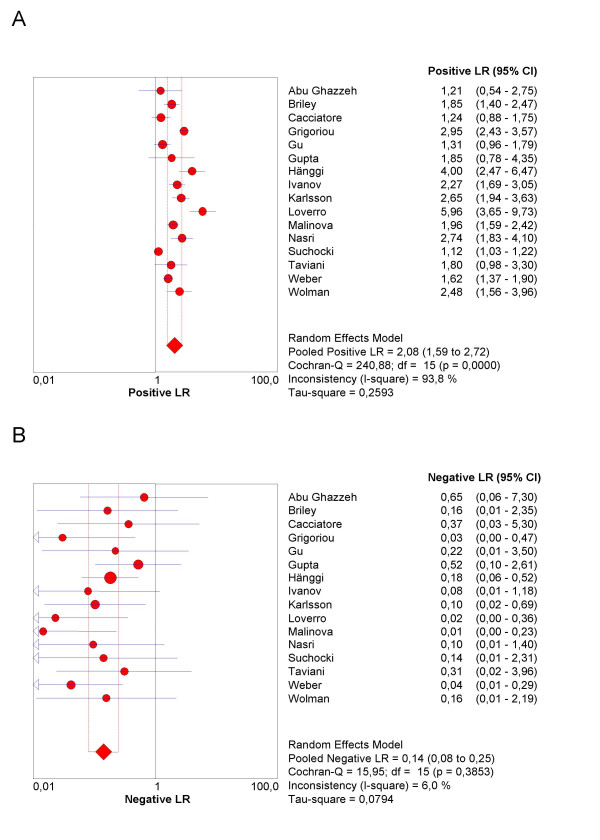
**Forrest plot**. Forrest plots of Likelihood ratios for positive (7a) and negative (7b) test results in one homogenous subgroup of studies of non-HRT users, with a test threshold of ≤ 5 mm, and using a single layer technique.

**Figure 8:**
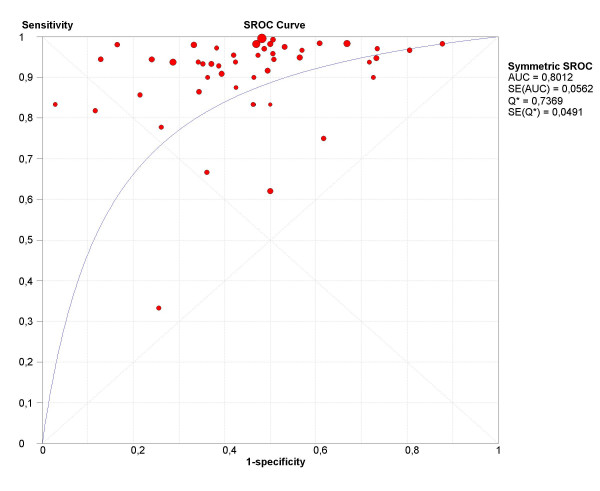
**sROC curve**. Receiver operating characteristics curve for all studies included in systematic review of ultrasound for prediction of endometrial cancer.

The final step in the analysis is pooling if this is considered appropriate. We illustrate pooling of the LRs for negative test results in one homogenous subgroup of studies of non-HRT users, with a test threshold of ≤ 5 mm, and using a single layer technique (Figure 7). Finally, we demonstrate sROC curve fitting in the presence of threshold effect for the whole data-set in Figure 8.

## Discussion and conclusion

Meta-DiSc allows description of individual study results; exploration of heterogeneity with a variety of statistics including chi-square, I-squared and Spearman correlation tests; implements meta-regression techniques to explore the relationships between study characteristics and accuracy estimates; performs statistical pooling of sensitivities, specificities, likelihood ratios and diagnostic odds ratios, using fixed and random effects models, both overall and in subgroups; and produces high quality figures, including forest plots and summary receiver operating characteristic curves that can be exported for use in manuscripts for publication.

Meta-DiSc is an evolving software. As new diagnostic meta-analytic methods become established over time, they will be implemented into the program in the future. For example, bivariate method of pooling sensitivity and specificity [18] is currently being developed. We will carefully follow the progress in this field. Once accepted as an established meta-analytic method, it will be implemented in Meta-DiSc. On similar lines, methods of data extraction from individual studies that only provide accuracy measures are currently being developed within our department. Once these methods have been verified, we will implement this option to assist systematic reviewers in extracting 2-by-2 tables from such studies.

Meta-DiSc is a comprehensive and dedicated test accuracy meta-analysis software. All computational algorithms in it have been validated through comparison with different statistical tools and published meta-analyses. Its use and citation in several meta-analyses published in high-ranking journals is evidence of external validation of its high quality [23-28].

## Availability and requirements

The software is publicly available at .

Operating system: The software runs on Windows based personal computers (Windows 95 or higher) with Pentium-class processor or equivalent, with minimum of 32 MB of RAM and minimum of 20 MB of hard disk space. SVGA color monitor; minimum 800 × 600 screen resolution and 256 colors.

Licence: Freeware for academic use.

## Competing interests

The author(s) declare that they have no competing interests.

## Authors' contributions

JZ conceived the idea. AM, VA and JZ developed the software. AC and KSK tested the software on a number of reviews and gave suggestions for improvements. All authors participated in preparing this manuscript.

## Pre-publication history

The pre-publication history for this paper can be accessed here:



## Supplementary Material

Additional File 1 Meta-Disc data set. This file contains simulated data. It is provided to help users to validate statistical procedures shown in table 1. Click here for file

Additional File 2 STATA data set. This file contains simulated data. It is provided to help users to validate statistical procedures shown in table 1. Click here for file
